# Editorial: XXXIII SIMGBM Congress 2019 - Environmental and Industrial Microbiology

**DOI:** 10.3389/fmicb.2021.640033

**Published:** 2021-03-23

**Authors:** Sara Borin, Margherita Sosio, Luigi Vezzulli, Carlo Viti

**Affiliations:** ^1^Department of Food, Environmental and Nutritional Sciences, University of Milan, Milan, Italy; ^2^Naicons Srl, Milan, Italy; ^3^Department of Earth, Environment and Life Sciences, School of Mathematical, Physical and Natural Sciences, University of Genoa, Genova, Italy; ^4^Department of Agricultural Science and Technology, Environmental Food and Forestry, School of Agriculture, University of Florence, Florence, Italy

**Keywords:** bioremediation, phytodepuration, antibiotic resistance, marine microrganisms, horizontal gene transfer, stone artwork consolidation, biomineralization, calcification

The Italian Society for General Microbiology and Microbial Biotechnology (SIMGBM) was founded in 1982. At present, the society has more than 350 members from Academia, Research Institutions, and Biotech Companies with the aim to foster microbiological research in Italy and to promote collaborations and visibility of Italian microbiologists in the different areas of microbiology and biotechnology. Articles presented in this Research Topic come from attendants of Microbiology 2019, the 33rd Congress of SIMGBM, held in Florence, Italy, on 19th−22nd June 2019 and include selected contributions under the label “Environmental and Industrial Microbiology.”

Microorganisms represent an environmental treasure in itself, playing a fundamental role in the sustainable development of ecosystems. Moreover, if exploited judiciously, they can be used to recover disturbed environments or for bioremediation processes. Both terrestrial and aquatic environments have been explored with this aim. In the papers from Vassallo et al. and Chamizo et al., microbial communities are studied aiming to obtain environmental benefits like the formulation of a synthetic bacterial community to improve phytodepuration processes, and for inoculation in soil restoration techniques, respectively. Vassallo et al. identified the dynamics of the microbiota composition of *Phragmites australis* roots as triggered by the presence of wastewater, identifying a direct correlation, linking the appearance of antibiotic- and synthetic wastewater-resistance with the time of exposure to wastewater. Cyanobacteria adapted to environmental stress can be used as inoculants to induce artificial biocrusts formation in degraded drylands. Exopolysaccharides (EPS) production is a key factor in biocrusts formation. In their study, Chamizo et al. investigated the differences in growth and polysaccharidic matrix features among three common biocrust-forming cyanobacteria proposed as soil inoculants. Although the proper care needs to be taken when releasing microorganisms into the environment, both studies highlight that a careful selection of microorganisms providing specific functions can be profitable for several environmental applications.

Selected microbial strains are, moreover, crucial to obtain specific biotechnological products. Three reviews are present in this Research Topic, focusing on three distinct applications of specific microbial groups, i.e., wild *Saccharomyces* strains for improved processes in industrial fermentations Di Paola et al., *Streptomycetes* for recombinant protein production (Berini et al. and calcium carbonate mineralizing bacteria for stone artwork consolidation (Marvasi et al.). The mini-review by Di Paola et al. outlines the history of fermentation and yeast domestication, leading to strain artificial selection by the breeding of wild species to obtain standardized yeast cultures. The authors highlight in particular the importance of social insects in yeast dispersion and in-gut breeding and discuss on their exploitation for the production of hybrid yeasts from environmental *S. cerevisiae* isolates suitable for industrial and biotechnological applications. The mini-review by Berini et al. focuses on the biotechnological exploitation of the streptomycetes by recapitulating recombinant protein production heterologously expressed in this genus in the last 40 years. The authors highlight *Streptomyces* as a promising, alternative, and versatile platform for recombinant protein production, discussing the pros and cons of using it as an expression chassis. Marvasi et al., finally, focus on the exploitation of bacteria to counteract the weathering threat to the stone cultural heritage given by pollution and global warming. Bacterial Calcium Carbonate Mineralization is indeed proposed as an environmentally friendly tool applicable *in situ* to protect calcareous stone artworks. The mini-review reports the milestones of the biomineralization approaches, discussing the challenging aspects and the perspectives of the different methods.

Marine Microbiology is a rapidly expanding branch of the science of Microbiology and thanks to latest generation molecular methodologies and technologies the nature, role, and biotechnological potential of marine microorganisms, the dominant life form on our planet, begin to be disclosed. In the work by Ottaviani et al. predatory bacteria belonging to the genus *Halobacteriovorax* were isolated from the Adriatic Sea and tested for their ability to prey pathogenic *Vibrio* spp. strains. Predator-prey interaction within the prokaryotic community was proposed to play a role in modulating the abundance of *V. parahaemolyticus strains* in the marine ecosystem through a top-down control of their bacterial prey community. Tamburini et al. investigate the structure and composition of the bacterial and archaeal communities in sediments from three Mediterranean ports. Using targeted metagenomic analysis of the 16S rRNA gene they were able to assess the effects exerted by multiple organic and inorganic contaminations on the benthic prokaryote community and designate bacterial community as a candidate tool for the monitoring of the sediment quality status in marine harbors.

The rise of extensive and widespread antibiotic resistance (AR) is one of the greatest threats to human health in environments exposed to antibiotic residues by means of wastewaters and animal manure. It has been recognized that some of the routes that cause AR spread in environments related to the agri-food system include the use of reclaimed water for irrigation purposes, and also the animal manure application to soils. In the study reported by Riva et al., the ability of an environmental *E. coli* strain, isolated form the crustacean *Daphnia* sp., to acquire exogenous DNA by natural competence with relatively high frequency was demonstrated. The protocol adopted was conceived to mimic conditions feasible in the environment, i.e., in natural and artificial water solutions considered as representative of environmental habitats. By also showing the capacity of this *E. coli* strain to colonize plant rhizosphere, using soil potted lettuce as a model system, the authors' results confirm the importance to investigate the possible spread of antibiotic resistant determinants through horizontal gene transfer in the environment and, particularly, in the rhizosphere of those employed plant species. Citterio et al. applied a PCR-based plasmid replicon typing to investigate the diversity and transferability of AR genes carrying plasmids in environmental *E. coli* strains, isolated from clams and marine sediments. Conjugative FIA, FIB, FII, plasmids (IncF group) were the most frequently found in AR strains isolated from the marine environment, suggesting a role played by these replicons in the spread of AR genes among environmental *Enterobacteriaceae* and, through the food chain, to human isolates.

Lastly, the use of antibiotics in the food animal industry is certainly considered among the main causes of propagation and dissemination of antibiotic residues, antibiotic-resistant bacteria (ARB) and antibiotic resistance genes (ARGs) in the soil-water system. The review by Checcucci et al. highlights the most recent research on ARGs in farm environment and the strategies used to control their dissemination. This review analyzes the most recent research on antibiotics and ARGs environmental dissemination conveyed by livestock waste. Strategies to control ARGs dissemination and antibiotic persistence were reviewed with the aim to identify methods for monitoring DNA transferability and environmental conditions promoting such a diffusion.

## Author Contributions

All authors contributed to the critical discussion of this Editorial and wrote and approved the final editorial.

## Conflict of Interest

MS was employed by company Naicons Srl. The remaining authors declare that the research was conducted in the absence of any commercial or financial relationships that could be construed as a potential conflict of interest.

